# Factors associated with treatment adherence to mandibular advancement devices: a scoping review

**DOI:** 10.1007/s11325-023-02862-9

**Published:** 2023-06-29

**Authors:** Liselotte H. van der Hoek, Boudewijn R.A.M. Rosenmöller, Liza J.M. van de Rijt, Ralph de Vries, Ghizlane Aarab, Frank Lobbezoo

**Affiliations:** 1 Department of Orofacial Pain and Dysfunction, Academic Centre for Dentistry Amsterdam (ACTA), University of Amsterdam and Vrije Universiteit Amsterdam, Amsterdam, The Netherlands; 2 Department of Oral and Maxillofacial Surgery, Amsterdam University Medical Centre, location Academic Medical Center (AMC), and Academic Centre for Dentistry Amsterdam (ACTA), University of Amsterdam and Vrije Universiteit Amsterdam, Amsterdam, The Netherlands; 3 Medical Library, Vrije Universiteit Amsterdam, Amsterdam, The Netherlands

**Keywords:** Adherence, Compliance, Mandibular advancement device, Obstructive sleep apnea

## Abstract

**Purpose:**

Obstructive sleep apnea (OSA) is frequently treated with continuous positive airway pressure (CPAP) or mandibular advancement devices (MADs). For various reasons, both treatment options are often affected by low adherence. While factors associated with low CPAP adherence are described in the literature extensively, less is known about adherence to MAD therapy. This scoping review aimed to synthesize the body of literature on the factors associated with adherence to MAD treatment.

**Methods:**

A systematic literature search was conducted using bibliographic databases PubMed, Embase.com, Web of Science, and the Cochrane Library (Wiley) to identify relevant studies that described factors associated with adherence to MAD in the treatment of OSA or snoring combined with OSA in adults.

**Results:**

The literature search yielded a total of 694 references. Forty studies were found eligible for inclusion. The literature showed that factors with a possible negative influence on the adherence to MAD treatment are personality aspects; failing effectiveness of MAD; side effects during MAD therapy; using a thermoplastic MAD; dental treatments during MAD therapy; and a poor first experience with the MAD with inadequate guidance by professionals. Factors that may have a positive effect on MAD adherence include effectiveness of therapy, custom-made MAD, good communication skills of the practitioner, early recognition of side effects, stepwise titration of the MAD, and positive first experience with MAD.

**Conclusions:**

The knowledge of factors associated with MAD adherence can be used to provide further insight into individual adherence to OSA treatments.

## Introduction

Obstructive sleep apnea (OSA) is a common condition that affects approximately 17% of women and 34% of men in the general adult population [1]. People with untreated OSA are at risk of diabetes type 2 [2], hypertension [3], and cardiovascular disease [4] and have a higher risk of traffic accidents [5]. Due to not only the high prevalence of OSA, but also personal health concerns and socioeconomic healthcare issues associated with OSA, effective treatment is essential.

The severity of OSA is determined by the apnea-hypopnea index (AHI) measured during a polysomnography (PSG). OSA severity is classified into mild (AHI ≥5 to <15), moderate (AHI ≥15 to <30), or severe (AHI ≥30) [6]. While the current standard treatment for patients with severe OSA is continuous positive airway pressure (CPAP) [7], oral appliance therapy is indicated for patients with mild to moderate OSA and for patients with severe OSA who cannot tolerate CPAP [8, 9]. Oral appliances advance the mandible and the tongue during sleep and thereby prevent obstruction of the upper airway. Such oral appliances are known as mandibular advancement devices (MADs). As neither CPAP nor MAD eliminate the underlying causes of upper airway collapsibility, lifelong treatment is necessary.

Although CPAP reduces OSA severity more than MAD therapy in patients with mild to severe OSA [10–12], its clinical effect is often compromised by low adherence [13, 14]. Adherence is commonly defined as behaving exactly according to rules or beliefs, which, in healthcare, usually involves conformity to treatment or medication [15, 16]. Another common related term is compliance, which is defined as obeying a particular law or rule or acting according to an agreement [15, 16]. Although the terms “compliance” and “adherence” are used synonymously, they are different in terms of the quality of healthcare, as the first term implies passively following of the instructions of the physician, while the second term implies active participation of the patient in the development of the treatment plan. The World Health Organization (WHO) prefers adherence to treatment over compliance [17]. Adherence to medical therapy is of utmost importance in the management of chronic conditions such as OSA. Studies on MAD therapy show varying results for long-term treatment adherence, which likely relate to many factors. Although numerous factors underlying poor adherence have been identified for CPAP therapy (Appendix 1) [13, 18–43], little is known about MAD adherence. Therefore, the aim of this scoping review was to assess the factors that may be associated with MAD adherence and to compare them with those related to CPAP adherence.

We hypothesized that patient-related factors associated with adherence to CPAP therapy — such as psychological status, perception of side effects, and social support [18, 33–36, 38] — also have an impact on adherence to MAD therapy. On the other hand, we also expected to find differences between adherence to both therapies regarding factors related to the device itself, such as effectivity and comfort. We expected to find that educational and behavioral interventions, as with CPAP [13, 26, 32], improve adherence to MAD.

## Methods

### Study design

This scoping review was conducted by the department of Orofacial Pain and Dysfunction of the Academic Centre for Dentistry Amsterdam (ACTA). The protocol was approved by the local ethics committee of ACTA (file number 2022-50457).

### Search strategy

On February 21, 2023, a literature search was performed in collaboration with a medical information specialist (RdeV) based on the Preferred Reporting Items for Systematic Reviews and Meta-Analyses extension for Scoping Reviews (PRISMA-ScR) statement (www.prisma-statement.org). To identify all relevant publications, systematic searches were conducted in the bibliographic databases of PubMed, Embase.com, Web of Science, and the Cochrane Library (Wiley). The following terms were used as index terms or free-text words (including synonyms and closely related words): “Mandibular Advancement” and “Treatment Adherence and Compliance.”

Wherever possible, the choice has been made to use MeSH terms in order to select only those articles that focused on our research aim. Duplicate articles were excluded using Endnote X20.0.1 (Clarivate™), following the Amsterdam Efficient Deduplication (AED)-method and the Bramer-method [44, 45]. For the full search strategies for all databases, see Appendix 2.

### Selection process

To establish whether the publications were relevant to our research aim, titles and abstracts were screened by a medical doctor studying for a dental Master’s degree (BR; MD, BSc) and a dental Master student (LH; BSc). The full-text articles were then checked for the eligibility criteria (see below). Differences in judgment were resolved during consensus meetings between BR, LH, and LR (DDS, PhD). To be included, a study had to describe factors that were associated with adherence to MADs in adults. Publications were excluded if they did not describe such factors and/or if the patients were under the age of 18. In addition, only articles that dealt with OSA or snoring combined with OSA were included. Further, all publication types were included, regardless of method. Only articles in English were included. The full texts of the selected articles were obtained for further review, in which positive and negative associations with adherence were examined.

## Results

**Fig. 1:**
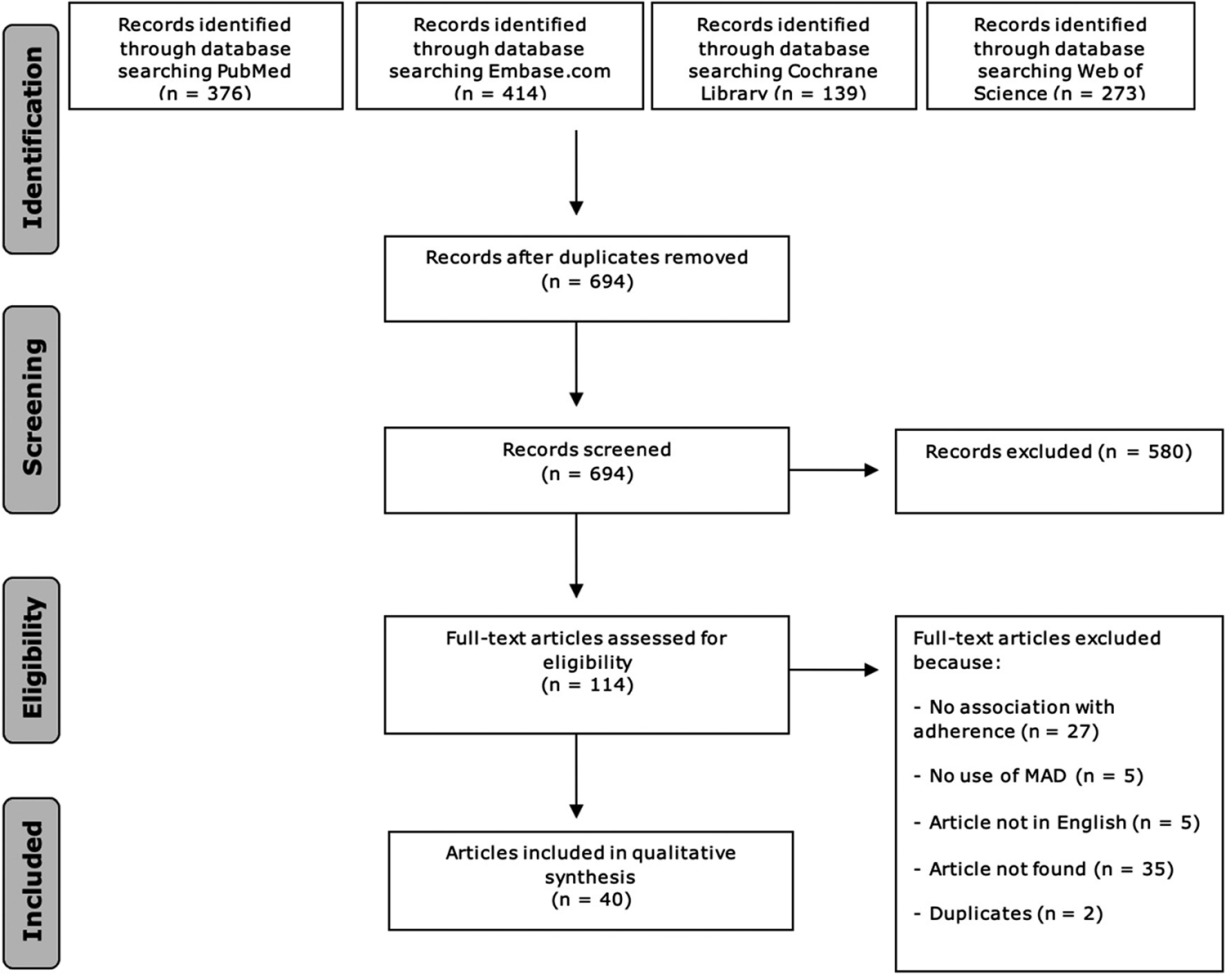
Flowchart of the search and selection procedure. *Note.* MAD, mandibular advancement device

**Fig. 2:**
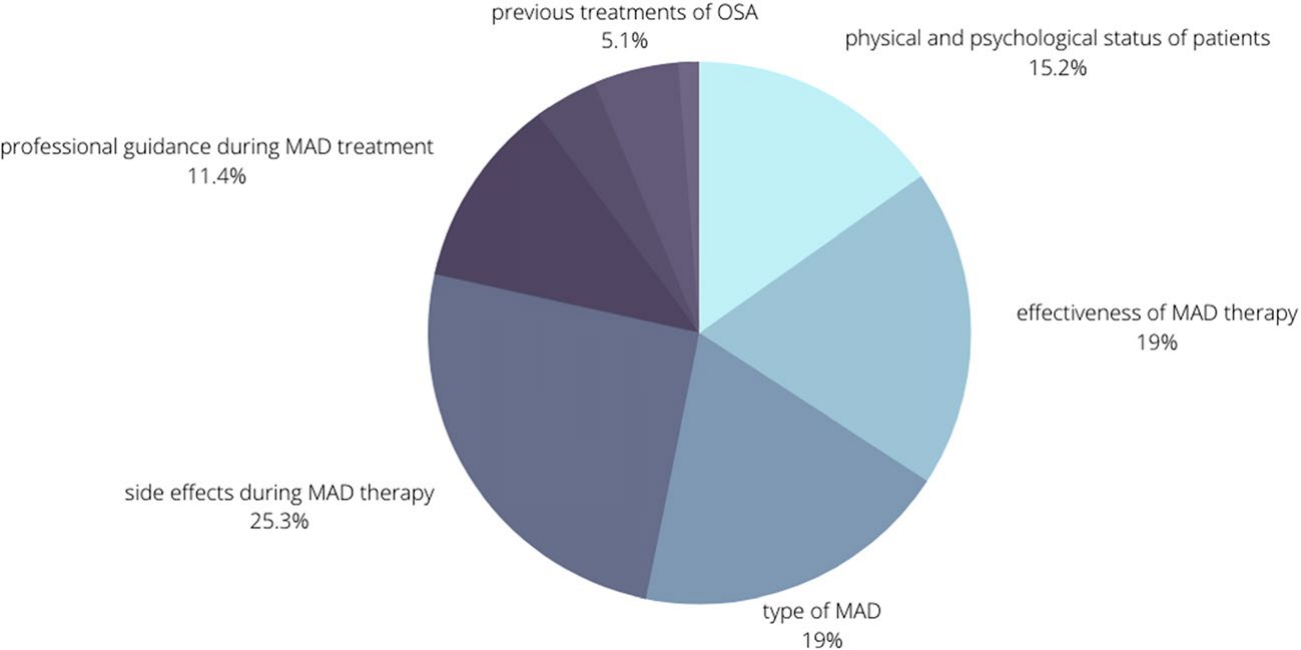
Frequency of adherence factors as described in the literature. *Note.* MAD, mandibular advancement device

The search resulted in 1202 articles (376, PubMed; 414, Embase.com; 139, Cochrane Library; 273, Web of Science). After removal of duplicate articles and screening of titles and abstracts, 114 articles remained for full-text screening (Fig. 1). Ultimately, we identified 40 primary studies for this scoping review that addressed factors associated with MAD adherence in adults. These studies were published between 2001 and 2022. The types of factors found to be associated with MAD adherence were grouped according to the following categories: physical and psychological status of patients, effectiveness of MAD therapy, type of MAD, professional guidance during MAD treatment, side effects during MAD therapy, dental treatments during MAD therapy, previous treatments of OSA, and quantity of factors of non-adherence. Table 1 gives an overview of all the included papers and which positive and negative adherence factors they have described. The column “categories” indicates under which subheading of the “Results” section the findings from the articles are classified. In the last two columns, we described how the data was obtained (based on scientific research (fact) or by expert opinion) and how the adherence was measured (objective, subjective (self-reported), or not applicable). Side effects during MAD therapy, effectiveness of MAD therapy, and type of MAD are the most frequently described factors related to adherence in the literature (Fig. 2).

**Table 1:** Overview of the characteristics of the included studies

Paper	Study design	Positive associations with adherence	Negative associations with adherence	Categories	Based on scientific research (fact) or expert opinion (opinion)	Measurement of adherence
Amoric, M. (2013)	Literature review	Communication skills of the practitioner	Occurrence of side effects, discomfort, pain, occlusal problems, and poor psychological disposition. Previously treated with another treatment modality	Physical and psychological status of patients, side effects during MAD therapy, professional guidance during MAD treatment, previous treatments of OSA	Fact + opinion	Not applicable
Attali, V. (2016)	Observational, single-center study	Good efficacy and tolerability of MAD. Reduction in AHI and complete symptom resolution, early follow-up. MAD therapy as a first line treatment	Relapse of nocturia	Effectiveness of MAD therapy, professional guidance during MAD treatment, previous treatments of OSA	Fact + opinion	Self-reported
Bachour, P. (2016)	Retrospective questionnaire study	Positive first experience, patients who were still using the device after 1 month. Perception of benefits and improvement of OSA symptoms	Side effects, treatment of snoring, backup for CPAP	Effectiveness of MAD therapy, side effects during MAD therapy	Fact + opinion	Self-reported
Bates, C.J. (2006)	Prospective cross-sectional cohort study	-	Initial side effects	Physical and psychological status of patients, side effects during MAD therapy	Fact + opinion	Self-reported
Berg, L.M. (2020)	Prospective, observational study based on data from a two-centered, parallel-arm randomized controlled trial (RCT)	-	-	Physical and psychological status of patients	Fact	Self-reported
Bortolotti F. (2022)	Systematic review with meta-analysis	Custom-made MAD, bi-block MAD.	Self-molded MAD.	Type of MAD	Fact	Self-reported
Bosschieter, P.F.N. (2022)	Single-center prospective randomized cross-over study	-	-	Type of MAD	Fact	Self-reported
Brette, C. (2012)	Retrospective cohort study	Patients with lower residual AHI or residual Epworth scores at month 3	Low success rate, occurrence of side effects, loss or breakage of the device. Number of interfering factors	Effectiveness of MAD therapy, physical and psychological status of patients, side effects during MAD therapy, type of MAD, quantity of factors of non-adherence	Fact	Self-reported
Chan, A.S. (2009)	Review	Custom-made MAD, early recognition of a lack of adherence and attention to symptoms of side effects	Self-molded MAD.	Type of MAD, professional guidance during MAD treatment	Fact + opinion	Not applicable
Cunali, P.A. (2011)	Double-blind, randomized, and controlled trial	Exercise support therapy in TMD patients with OSA	TMD pain.	Physical and psychological status of patients, side effects during MAD therapy	Fact	Self-reported
De Ruiter, M.H.D. (2020)	Observational intervention trial part of a randomized controlled trial	More protrusion of the mandible	Patients who declined more protrusion	Professional guidance during MAD treatment	Fact	Objective
Deacon, N.L. (2016)	Review	Early recognition of a lack of adherence and attention to symptoms of side effects. Positive first experience	-	Professional guidance during MAD treatment, side effects during MAD therapy	Fact	Not applicable
Dieltjens, M. (2012)	Descriptive survey design	Bi-block MAD	Type D personality, mono-block MAD	Physical and psychological status of patients, type of MAD	Fact	Self-reported
Dieltjens, M. (2015)	Prospective clinical study	Improvement of OSA symptoms/reduction in snoring	When no change was noticed by the patient or partner, occurrence of side effects	Effectiveness of MAD therapy, side effects during MAD therapy	Fact	Objective
Dioguardi, A. (2016)	Review	Patient has a part in choosing the device	-	Side effects during MAD therapy, professional guidance during MAD treatment	Fact + opinion	Not applicable
Friedman, M. – 2012	Retrospective review of data collected from 2 nonrandomized, noncontrolled parallel series	Custom-made MAD	Self-molded MAD, occurrence of side effects, previously treated with another treatment modality	Type of MAD, side effects during MAD therapy, previous treatments of OSA	Fact	Self-reported
Gagnadoux, F. (2017)	Prospective nonrandomized study	-	Occurrence of side effects	Type of MAD, side effects during MAD therapy	Fact	Self-reported
Gjerde K. (2022)	Prospective study	Effectiveness of MAD therapy, positive partner perceptions	Side-effects	Effectiveness of MAD therapy, side effects during MAD therapy	Fact	Objective
Haviv, Y. (2017)	Retrospective chart review study	-	Effects of the oral appliance on teeth, insufficient efficacy, discomfort or improved well-being following weight loss. Three main parameters which negatively impact long-term adherence: low subjective efficacy, smaller objective success rate, and a high number of interfering factors. Dental treatments during MAD therapy	Effectiveness of MAD therapy, side effects during MAD therapy, dental treatments during MAD therapy, quantity of factors of non-adherence	Fact	Self-reported
Hoffstein, V. (2007)	Review	-	Discomfort, perception of little or no benefit, low success rate	Effectiveness of MAD therapy, side effects during MAD therapy	Fact	Not applicable
Ingman, T. (2013)	Retrospective review	Both maxillary and mandible lengths, i.e., the shorter the mesio-distal lengths of maxilla and/or mandible were, the better the patient adapted to the MAS. Retrognathic position of maxilla	-	Physical and psychological status of patients	Fact	Self-reported
Jacobowitz, O. (2017)	Review	Comfort, nasal patency, and effectiveness	Occurrence of side effects.	Side effects during MAD therapy	Opinion	Not applicable
Johal, A. (2018)	Systematic review	Custom-mad MAD.	Ready-made MAD (lack of retention).	Type of MAD	Fact	Self-reported
Johal, A. (2017)	Randomized crossover trial	Custom-made MAD.	Ready-made MAD.	Type of MAD	Fact	Self-reported
Kwon J.S. (2022)	Randomized, prospective and controlled crossover design	Feedback services of MAD	-	Professional guidance during MAD treatment	Fact	Objective
Lee, W. H. (2013)	Retrospective clinical trial	Bi-bloc MAD.	Mono-bloc MAD	Type of MAD	Fact	Self-reported
Liu J. (2021)	Randomized Controlled Trial	Multifactorial intervention	-	Professional guidance during MAD treatment	Fact	Objective
McGown, A. D. (2001)	Retrospective questionnaire study	Snoring and symptoms subjectively improved, patients with daytime symptoms	Discomfort, lack of effectiveness, a higher average number of side effects. No change noticed by the patient or their partner	Effectiveness of MAD therapy, side effects during MAD therapy	Fact	Self-reported
Nerfeldt, P. (2016)	Prospective intervention cohort study	Arousers (women).	Desaturaters (higher BMI)	Effectiveness of MAD therapy, Physical and psychological status of patients	Fact	Self-reported
Pahkala R. (2021)	Prospective study	Reduction in snoring	Mandibular retrusion, bruxism, and daily smoking	Effectiveness of MAD therapy, Physical and psychological status of patients	Fact	Objective
Pépin, J. L. (2019)	Prospective, multicenter, randomized, controlled, open trial	Custom-made MAD	Self-molded MAD	Type of MAD	Fact	Self-reported
Prescinotto, R. (2015)	Prospective study	Lower values of AHI and arousals at baseline	Patient discomfort, occurrence of side effects. Number of interfering factors	Effectiveness of MAD therapy, Physical and psychological status of patients, side effects during MAD therapy, quantity of factors of non-adherence	Fact + opinion	Self-reported
Quinnell, T. G. (2014)	Open-label, four-period, crossover, randomized controlled trial	Custom-made MAD.	Poor retention, self-molded MAD	Type of MAD	Fact	Self-reported
Saglam-Aydinatay, B. (2018)	Retrospective questionnaire study	Younger age, effectiveness of MAD therapy, ease of use, support from their partner, the shame caused by disease symptoms and portability of the appliance	Older age, inability to adapt to the appliance, pain in the TMD joint, ineffectiveness in decreasing symptoms, dry mouth	Effectiveness of MAD therapy, Physical and psychological status of patients, side effects during MAD therapy	Fact	Self-reported
Sutherland, K. (2021)	Review	-	Therapeutic infectivity, inefficacy, tooth discomfort or pain, difficulty sleeping with the appliance, odontologic problems, type D personality patients, initial side effects	Physical and psychological status of patients, effectiveness of MAD therapy, side effects during MAD therapy	Fact	Not applicable
Sutherland, K. (2021)	Prospective study	Initial treatment period of 20 days.	Lack of OSA symptoms at start of therapy	Side effects during MAD therapy, effectiveness of MAD therapy	Fact	Objective
Tallamraju, H. (2021)	Systematic review with meta-analysis	Custyom-made MAD	Side effects	Type of MAD, side effects during MAD therapy	Fact	Self-reported and objective
Uniken Venema, J.A.M. (2021)	Systematic review with meta-analysis	Custom-made MAD	Self-molded MAD	Type of MAD	Fact	Self-reported
Vanderveken, O. M. (2008)	Randomized controlled cross-over trial	Custom-made MAD	Self-molded MAD	Type of MAD	Fact	Self-reported
Vuorjoki-Ranta, T. R (2020)	Retrospective questionnaire study	Reduced loud snoring, multidisciplinary medical approach, regular control visits. CPAP or surgery treatment in the past	Setbacks of disease, low success rate	Effectiveness of MAD therapy, professional guidance during MAD treatment, previous treatments of OSA	Fact + opinion	Self-reported

*MAD* mandibular advancement device; *AHI* apnea-hypopnea index; *OSA* obstructive sleep apnea; *CPAP* continuous positive airway pressure; *TMD* temporomandibular disorder

### Quality assessment and reliability

This scoping review is intended to provide an overview of all existing evidence, regardless of quality. Therefore, a formal assessment of the methodological quality of the included studies was not performed [46]. Nevertheless, to give an indication of the quality of the included studies, the factors found were assessed for quality by distinguishing between scientifically researched or expert opinion. The distinction between the two categories was made by the way in which the described data was obtained and presented in the included studies. Whereas 2.5% of the factors derived from expert opinion, 77.5% of the factors were on scientific research, and the remaining 20% were based on both. Additionally, the method of measuring adherence was examined. Where possible, a distinction was made between objective or subjective (self-reported). Of the 40 articles included, seven measured adherence objectively, 25 measured it subjectively (self-reported), one article measured adherence both subjectively and objectively, and seven articles did not mention this (not applicable). Also, of all 40 included studies, seven articles are randomized controlled trials (RCT) and two articles are systematic reviews and meta-analysis [47]. The remaining articles are reviews, cohort studies, and expert opinions.

### Factors associated with MAD adherence

The results are divided into several categories. For each category, the results are noted.

#### Physical and psychological status of patients

It is important to look at the patients’ physical characteristics before starting MAD therapy. Among OSA patients, some are arousers, i.e., patients who wake up at night, and others are desaturaters, i.e., patients who have low saturation during the night. There is a significant difference in MAD adherence between arousers (85%) and desaturaters (55%). Women were classified more as arousers than men, and patients with a higher body mass index (BMI) had more oxygen desaturations than those with a lower BMI [48]. Patients of younger age were more adherent than older-aged subjects in the treatment of mild or moderate OSA [49], and patients familiar with bruxism or smoking on a daily basis were less adherent [50].

Looking at anatomic features, Berg et al. concluded that even though tongue size of patients may predict OSA severity, no association was found between Friedman score (i.e., a score developed to describe and classify the morphology of the oropharynx with the tongue in a natural relaxed position [51]) and treatment adherence in patients with mild to moderate OSA receiving MAD treatment [52]. Although Ingman et al. found positive correlations between maxillary and mandible mesio-distal lengths and a trend toward a retrognathic maxilla position in compliant patients [53], Prescinotto et al. found no influence of anatomical features on treatment adherence [54], in contrast, Pahkala et al. found that mandibular retrusion negatively affects adherence [50]. Patients familiar with a temporomandibular dysfunction (TMD) diagnosis prior to MAD treatment and diagnosed with mild to moderate OSA adhered better to MAD therapy when they got exercise support therapy during their MAD therapy in the first 120 days of their treatment [55]. Other studies described no significant association between patient phenotype (e.g., sex, age, education, anthropometric characteristics, and economic status) and adherence rates [54, 56–58].

Also, certain personality traits have been linked to poor adherence. In medical psychology, personalities are often divided into 4 types of personalities, from A to D. Type D personality is defined as the simultaneous presence of both negative affectivity and social inhibition. Therefore, the letter D stands for distressed [55]. Type D personality may be related to the fact that these patients experience a higher frequency and extent of side effects, which reduces their confidence in treatment and affects MAD adherence. Research has shown that type D patients reported a significantly lower adherence to MAD treatment and exhibit a higher discontinuation rate when compared to patients without a type D personality [47, 59]. Also, a poor psychological disposition (i.e., perception of their state of health, and their mental state measured by a depression test) had an association with poor MAD adherence [60].

#### Effectiveness of MAD therapy

Therapy effectiveness is one of the most important reasons for adherence and continuation of therapy. In Attali et al., it was described that a ≥50% reduction in AHI and a complete symptom resolution at short-term evaluation had a positive association with adherence and is a significant predictor for long-term MAD usage [61]. Similarly, an improvement of OSA symptoms was observed with long-term MAD adherence for OSA therapy [62]. This was also described in Dieltjens et al., who found that a reduction in snoring is perceived as a therapeutic effectiveness and therefore promotes the use of the MAD, thereby improving adherence [63]. Noticing snoring and apneas by the bed partner is positively associated with adherence, so information from the bed partner about reduction in breathing cessations can support patients’ MAD adherence [64]. While treatment adherence increased in patients whose snoring and daytime OSA symptoms subjectively improved, patients tended to stop treatment when no change was noticed, either by themselves or by their partner [63, 65]. There is a significant difference in MAD adherence between arousers (85%) and desaturaters (55%) (see paragraph 3.2.1: “Physical and psychological status of patients”) [48]. Patients with mainly arousals at their PSG were more adherent at 12 months of therapy, as opposed to patients with more oxygen desaturations at their PSG. Also, a lower value of AHI and less arousals at baseline are associated with better adherence. This suggests that patients with milder OSA have better adherence to MAD treatment than those with severe OSA [54]. Furthermore, Bachour et al. described that high adherence could be due to a strict MAD indication policy, not starting MAD therapy in simple snorers or as a backup or additional therapy to CPAP [62]. The absence of OSA symptoms at the start of therapy negatively affects MAD adherence [66], while Pahkala et al. found that on baseline, the presence of a more pronounced disturbance of snoring correlates positively with objective adherence [50]. The main reason for discontinuation of the treatment was inefficacy of the treatment [47, 49, 61, 65, 67]. It is described that both a lower objective success rate (e.g., higher residual AHI with the device) and a lower subjective success rate (e.g., more residual sleepiness or relapse of symptoms, still snoring, or the partner noticed no change) are the reasons for discontinuation [47, 57, 65, 67–69].

#### Type of MAD

In general, the MADs currently on the global market can be divided into 2 categories: self-molded and custom-made. A self-molded thermoplastic MAD must usually be warmed up with hot water and then placed in the mouth so that it molds itself to the patient. In contrast, a custom-made MAD is manufactured by professionals. The existing literature shows a great preference and better adherence rates for custom-made MADs, mainly because of their better retention [58, 70–78]. Only Bosschieter et al. found similar adherence for both MADs [79]. In addition, custom-made MADs performed more favorable than self-molded thermoplastic MADs in AHI reduction [11, 80]. Overall disadvantages of thermoplastic designs include more side effects, difficulties in tolerating the device, and a lack of retention, resulting in poor adherence rates. However, the possibility of home-based fitting and its lower costs are advantages [80]. Another distinction can be made between mono-bloc or bi-bloc MADs. Mono-bloc MADs consist of one part, while bi-bloc MADs consist of two separate parts. While the efficacy of a mono-bloc MAD is greater because it allows no movement of the jaw, the relatively free movement of the jaw when using bi-bloc MAD may be the explanation for the better adherence of bi-bloc MAD compared to mono-bloc MAD [59, 78, 81]. Another common reason for discontinuing MAD therapy was loss or breakage of the device and possible cost of replacement [57].

#### Professional guidance during MAD treatment

Regular follow-up of patients by a sleep physician and a dental practitioner is required to monitor the treatment response, side effects, and adherence. In order to increase adherence, communication skills of the practitioner and early recognition and attention to symptoms of side effects are important [60, 69, 70, 82]. Attali et al. suggested that, as with other chronic diseases, early follow-up of OSA patients (e.g., repeating PSG) is very important because short-term efficacy is strongly predictive for continuation of MAD therapy [61]. Additionally, patients are more likely to be adherent to a device when they have participated in the decision-making process [83]. Kwon et al. revealed that an increase of objective adherence with MAD was seen when using feedback services by information and communication technology (ICT) [84]. A multifactorial intervention (e.g., additional information for the patient’s partner) in addition to standard MAD care improved patient adherence to treatment as well [85].

De Ruiter et al. found that stepwise titration and more protrusion of the mandible result in good adherence [86]. A possible explanation for this could be that patients are motivated to receive optimal treatment but want to experience as few side effects as possible. Patients who declined more protrusion of the mandible were less adherent to treatment. Even though there is a relationship between increased advancement of the mandible and better therapeutic efficacy, increased advancement may increase TMD risk, thereby affecting adherence negatively [47, 87].

#### Side effects during MAD therapy

Common side effects with MAD treatment include excessive salivation, xerostomia, occlusal problems, dental discomfort, jaw discomfort, and gingival discomfort. The occurrence of these side effects is often associated with low adherence [50, 55, 58, 59, 61, 64–66, 68, 69, 75, 78, 83]. Nevertheless, the side effects of MADs are usually mild by intensity and are not an obstacle to long-term regular use [84]. Also, TMD pain is a prevalent reason for low adherence [50, 56]. TMD pain could already have been present before starting the MAD therapy or have arisen in relation to the use of the MAD. Mandibular exercises were found to be effective in reducing pain and increasing MAD adherence [56]. Adherence to MAD depends, among other things, on the balance between perceptions of benefit and side effects [63, 68]. Although persistent side effects are rare [85], initial side effects may prevent early acceptance of the MAD and contribute to non-adherence [48, 57].

Positive initial experiences with MAD treatment predict increased long-term adherence [62, 82]. Sutherland et al. suggest that the first 20 days of the initial treatment period is important to perform interventions in patients with an expectation of poor treatment adherence [66]. The long-term adherence rate was high in patients who were still wearing their MAD after one month of starting their MAD therapy [62]. There could be a correlation between the initial effects of the therapy and the additional side effects. The presence of side effects such as dry mouth and discomfort were reasons for stopping treatment within 6 months. Patients who discontinued after longer use of the MAD mainly reported that it was due to occlusal changes [88]. Therefore, initial side effects could be a reason for early treatment failure, and it could prevent adherence to therapy [56].

#### Dental treatments during MAD therapy

Dental treatments in patients during MAD treatment was a major reason for discontinuation of MAD therapy, mainly because of an inaccurate fit of the MAD after adding supplementary occlusal units (i.e., dental implants, dental bridges) or making changes to the shape of the teeth and/or molars. In addition, patients became worried that more dental problems would arise in the future or that new dental restorations could be damaged due to the use of the MAD [67].

## Previous treatments of OSA

Sometimes, patients with OSA start with another treatment modality before being referred for MAD therapy. When patients are previously treated with another treatment modality, they come from a situation of treatment failure, so Amoric et al. stated that there is a likelihood this category of patients will be more inclined to abandon this new treatment as well, because of their poor psychological acceptance ability [60]. Friedman et al. found that adherence to a custom (50.9%) or a self-molded (32.5%) MAD was poor after 6 months, but explained that this adherence rate was acceptable because all patients in this study had previously failed OSA therapy (e.g., CPAP and/or upper airway surgery) [74]. MAD-therapy as a first-line therapy was one of the predictors of long-term use of MAD [61]. In contrast to this conclusion, Vuorjoki-Ranta et al. found that many of their patients were still using their MAD after receiving CPAP therapy or surgery 5 years earlier [69].

### Quantity of factors of non-adherence

Non-adherence to MAD therapy is influenced by many of the factors described above. The amount of these factors associated with non-adherence affects patient adherence. The more possible factors for non-adherence are present, the higher the likelihood of actual non-adherence [54, 57, 67].

## Discussion

This scoping review aimed to identify the various factors associated with MAD adherence and compare them to the factors associated with CPAP adherence. The available literature described that several factors may have an association with adherence to MAD treatment. Factors that may have a positive impact on MAD adherence include good effectiveness of therapy, custom-made MAD, good communication skills of the practitioner, early recognition of side effects, stepwise titration of the MAD, and positive first experience with MAD. Main factors with a possible negative influence on the adherence to MAD treatment are personality aspects like a type D personality; failing effectiveness of MAD; occurrence of side effects during MAD therapy, such as patient discomfort or dental pain; usage of a thermoplastic MAD instead of a custom-made one; dental treatments during MAD therapy; and a poor first experience with the MAD with inadequate guidance by professionals. Side effects during MAD therapy, effectiveness of MAD therapy, and type of MAD are the most frequently described factors related to adherence in the literature (Fig. 2). This does not imply that these factors in fact have the greatest influence on adherence. Therefore, in this scoping review, all potential factors were examined.

A distinction can be made between MAD-specific factors and general factors of OSA therapy adherence. MAD-specific factors include occurrence of side effects during MAD therapy; the type of MAD; and dental treatments during MAD therapy. General factors include personality aspects of patients; failing effectiveness of treatment; poor experience of initial treatment with inadequate guidance by professionals; and poor experience with previous treatment modalities. The distinction between both factors is relevant for the purpose of achieving patient’s adherence.

As to enable the comparison between factors influencing adherence to MAD and to CPAP, a search was performed in PubMed with the aim to retrieve factors known to influence adherence to CPAP from systematic reviews (see Appendix 1). Patients with mild OSA are less likely to be compliant with CPAP therapy than patients with moderate to severe OSA[31]. Also, Holley et al. found that there was no significant difference in mild OSA patients between CPAP and MAD treatment in achieving their target AHI [89]. The factors listed above associated with MAD adherence in the treatment of OSA indicate that there are both similarities and differences in physical and psychological status of patients, type of device, and professional guidance during treatment between MAD and CPAP. Both treatments cause different side effects, but for each device, a high frequency or severity of side effects negatively affects adherence [18, 60]. Unlike CPAP, in the case of MAD treatment, TMD is also a reason for poor adherence [55], although a case report has described TMD-related side effects in association with CPAP as well [90]. It is noteworthy that patients with mild OSA are likely to have better adherence with MAD, and patients with severe OSA with CPAP [31, 54]. Lack of support from a partner or family, when no change is noticed by them, has a negative impact on adherence with both MAD and CPAP [18, 36]. Factors related to psychological status also affect both treatments. For example, in both CPAP and MAD treatment, type D personality and a poor perception of their general and mental state of health are of influence [38, 47, 59, 60]. However, for CPAP only, a psychological comorbidity, such as PTSD, influences adherence [33–35, 91, 92]. To our knowledge, however, this has not yet been researched for MAD in the current literature. When looking at device-related factors in MAD and CPAP therapy, for both treatments, the design of the device is a topic that has been extensively studied and can affect adherence. For example, for CPAP, the type of mask is of influence, while for MAD, there is a difference between custom-made or thermoplastic devices [11, 20, 21, 64, 80, 93]. For both CPAP and MAD, adherence is improved when using feedback services by ICT [21–25, 43, 84]. It is important to improve and enhance these technologies in the future. Also, MAD and CPAP adherence are both affected by the practitioner in terms of their communication skills or the use of behavioral interventions [13, 18, 26, 32, 60, 64, 69].

Although many interventions have been described in the literature on improving CPAP adherence, such as behavioral interventions like motivational interviewing, few interventions have been described for improving MAD adherence [18]. However, Dioguardi et al. described that patients who are included in the decision-making process of choosing a therapy have better treatment adherence [83]. Also, De Ruiter et al. described that stepwise titration of protrusion of the mandible improves MAD adherence [86].

Only 40 articles were included in this study, despite including all publication types being accepted. Therefore, our findings for this scoping review indicate a scarcity of literature specifically addressing MAD adherence in OSA therapy. Clearly, more research is needed to adequately reveal the associated factors with MAD adherence.

When looking at the type of MAD, it is noteworthy that Uniken Venema et al. described that mono-bloc appliances, compared to bi-bloc appliances, performed more favorable when examining AHI reduction. However, there were no clinically relevant differences in reduction of the Epworth Sleepiness Scale (ESS), preference, side effects, and cost-effectiveness, and in contrast to Dieltjens et al. and Lee et al., in the study by Uniken Venema et al., there were no differences between mono-bloc or bi-bloc MADs in adherence [59, 80, 81].

When comparing our scoping review to the systematic review and meta-analysis of Tallamraju et al., both studies found no significant correlation between adherence and patient or disease characteristics. Patients who did not adhere to the therapy reported experiencing more side effects and were more likely to discontinue the treatment within the first three months. Additionally, custom-made oral appliances were found to be preferred and associated with higher adherence compared to self-molded appliances. However, further research is needed to investigate the relationship between psychosocial factors and adherence to oral appliance therapy [58].

### Strengths and limitations

To summarize the existing literature on factors associated with MAD adherence, 4 different databases were searched (PubMed, Embase.com, Web of Science, and the Cochrane Library) with guidance of a medical information specialist. The selection of suitable articles took place in a structured manner by two independent researchers with the help of another researcher to settle differences in judgment. This selection procedure ensures high reproducibility and thus low bias. Only articles in English were included in this scoping review leaving out 5 potentially eligible articles. As a result, additional data may have been missed. To compare the identified factors associated with MAD adherence with those associated with CPAP adherence, a limited search was performed in one database, viz., PubMed, in which only systematic reviews were included. This was done mainly because compared with MAD, there are a substantial number of studies on CPAP adherence. Although only one database was searched, it may be assumed that no important factors were missed because systematic reviews were included, in which the factors have already been mapped. Also, among the identified factors associated with MAD adherence in this scoping review, not all have been scientifically studied. Of these factors, 2.5% were derived from expert opinion, 77.5% from scientific research, and the remaining 20% from both. Thus, some of the factors are not based on scientific research which may cause a biased outcome. Considering that most studies relied on subjective adherence assessments, this scoping review observed a lack of objective measurement of adherence. Although there is a high correlation between subjective and objective data, subjectively measured adherence can cause a biased outcome because it tends to overestimate the actual MAD use [94, 95]. This suggests that more studies that objectively assess adherence are necessary.

### Clinical implications

The various factors associated with professional guidance in MAD therapy emphasize the importance of regular follow-up consultations, early follow-up, including the patient in the decision-making process, and performing stepwise titration [60, 61, 69, 70, 82, 83, 86]. These results highlight the importance of integrated, interdisciplinary care between sleep physicians and dental practitioners with regular follow-up to ensure the long-term adherence to MAD treatment [96].

A clinician should develop a clear path of care for OSA patients treated with MADs. Patients should be informed at intake with a clear introduction about the MAD regarding a habituation phase and side effects in the short- and long-term of their MAD treatment. Regular appointments ensure proper monitoring of MAD treatment and for patients to report side effects or other discomforts.

MAD as a first-line treatment facilitates better long-term use [62]. Patients with previous treatment failure might be extra motivated, properly followed up, and instructed at the start of their therapy to make the new treatment a success [70]. Because patients with previous treatment failure may have poor psychological acceptability, these patients will likely stop with this new treatment as well [61, 75].

In addition, when starting MAD treatment, it is important to check the dental situation by a dental specialist, because short-term dental treatments are a common problem with the fit of the MAD [67]. In these situations, MAD therapy should be postponed until a stable and healthy dental situation is reached. Also, the patient should always bring their MAD with them to the dental practitioner. Should any adjustments be made to the dentition, the MAD may then be adjusted by the dental professional as needed.

Also, improved patient adherence was noticed with custom-made MADs compared to thermoplastic self-molded MADs; therefore, custom-made MADs are recommended [80]. Future research could be done to optimize the thermoplastic MAD design by improving retention and, consequently, realizing better therapy adherence.

## Conclusion

This scoping review described several factors that may have an association with adherence to MAD therapy, such as physical and psychological status of patients, effectiveness of MAD therapy, type of MAD, side effects during MAD therapy, professional guidance during MAD treatment, dental treatments during MAD therapy, previous treatments of OSA, and quantity of factors of non-adherence. When comparing the factors associated with MAD adherence to those associated with CPAP adherence, there are both similarities and differences in the categories physical and psychological status of patients, type of device, and professional guidance during treatment. These results show that MAD adherence does not depend on the same factors as CPAP adherence, but there is some overlap.

The knowledge of the factors found in this study associated with MAD adherence can be used for further research to make recommendations to improve adherence. Ultimately, further studies would be useful to detect non-adherent patients before starting treatment and to provide proper guidance in the early stages of treatment. Matching patients to treatments in a personalized manner may contribute to the efficacy of OSA management. To facilitate clinical decision-making, more prospective randomized studies are needed.

## Figures and Tables

**Fig. 3:**
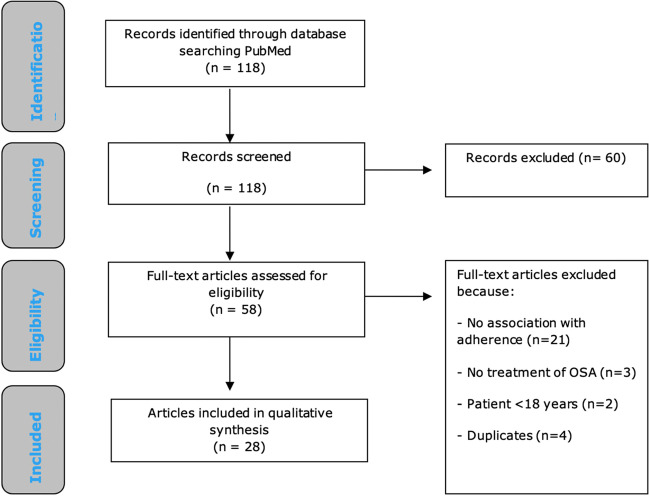
Flowchart of the search and selection procedure of reviews describing factors associated with CPAP adherence. *Note.* OSA, obstructive sleep apnea

## Data Availability

The authors declare that the data supporting the findings of this study are available within the article.

## Appendix 1

### Literature review

To ultimately compare the factors associated with MAD adherence with those associated with CPAP adherence, it was first necessary to identify them. To that end, a literature search on systematic reviews on factors associated with CPAP adherence was conducted in the bibliographic database of PubMed on May 9, 2021.

### Search strategy

To identify all relevant publications, the following MeSH terms were used: “Treatment Adherence and Compliance,” “Continuous Positive Airway Pressure,” and “Systematic Review.” Systematic reviews that described factors related to CPAP adherence in the treatment of OSA in adult patients were included. The search resulted in 118 articles. After screening for title and abstract, 24 articles remained. Several factors related to CPAP adherence emerged after reading the full text articles.

### Selection process

To determine if the publications were relevant to our research aim, titles and abstracts were screened by a medical doctor studying for a dental master’s degree (BR; MD, BSc) and a dental master’s student (LH; BSc). The full-text articles were then checked for eligibility criteria, and differences in assessment were resolved during consensus meetings between BR and LH. To be included, a systematic review had to describe factors associated with adherence to CPAP in adult OSA patients. Publications that did not describe such factors and in which the patients were younger than 18 years were excluded. Only systematic reviews were included.

### Results

The search resulted in 118 articles. After screening of the titles and abstracts, 24 articles remained for full-text screening (Fig. 3). All factors associated with CPAP adherence were noted. The types of factors found to be associated with CPAP adherence were grouped according to the following categories: physical and psychological status of patients, type of CPAP, professional guidance during CPAP treatment, and medication use during CPAP treatment.

### Physical and psychological status of patients

Patient-related factors that influence adherence to CPAP include disease and patient characteristics, psychological factors, and social factors [18]. Patients with mild OSA are less likely to be compliant with CPAP therapy than patients with moderate to severe OSA [31]. Patients who had upper airway surgery showed improved CPAP adherence [37], and nasal anatomy may be of influence on CPAP adherence [18]. When looking at psychological factors, it was found that patients with post-traumatic stress disorder (PTSD) showed low adherence to CPAP and that improvements in PTSD symptoms were associated with better CPAP adherence [33–35]. Adults with type D personality (i.e., negative affectivity and social inhibition) perceived more side effects and showed lower CPAP adherence than patients without type D personality [38]. Proper family and social support are an important factor in experiencing CPAP treatment; this requires further study to determine the impact it has on CPAP adherence [36].

### Type of CPAP

It was notable that many factors associated with CPAP adherence are device related. For example, CPAP adherence is influenced by the type of mask, being higher with nasal masks than with oronasal masks [19–21]. Looking at pressure delivery, some studies reported no significant difference between auto-CPAP and conventional CPAP in adherence [27, 28, 41], while others showed better results for auto-CPAP [26, 29, 42, 97]. Although no significant difference in adherence is observed when the humidification of the device is adjusted, heated humidification reduces some CPAP-related side effects more than no humidification [30].

### Professional guidance during CPAP treatment

Improvement of CPAP adherence is shown with educational and behavioral interventions [13, 26, 32] as well as with telemonitor interventions [21–23, 25]. Also, the use of patient-facing applications (i.e., web-based applications that interact directly with the patient) improved adherence [43], and eHealth interventions increase adherence during the initial phase of treatment [24].

### Medication use during CPAP treatment

When examining CPAP adherence and the use of non-benzodiazepine sedative hypnotics, it shows that such hypnotics may improve CPAP adherence [39]. The same observation applies to the use of nasal steroids [40].

## Appendix 2

**PubMed session results (21 Feb 2023)**

**Table:**

Search	Query	Items found
#3	**#1 AND #2**	376
#2	**“Treatment Adherence and Compliance”[Mesh] OR complian*[tiab] OR adheren*[tiab] OR noncomplian*[tiab] OR nonadheren*[tiab] OR dropout*[tiab] OR “drop-out*”[tiab]**	575,979
#1	**“Mandibular Advancement”[Mesh] OR ((“mandibular advancement”[tiab] OR “maxillomandibular advancement”[tiab] OR “mandibular reposition”[tiab]) AND (device*[tiab] OR appliance*[tiab])) OR “protrusive device*”[tiab] OR “protrusion device*”[tiab] OR “protrusive appliance*”[tiab] OR “protrusion appliance*”[tiab]**	2,561

**Embase.com****session results (21 Feb 2023)**

**Table:**

Search	Query	Items found
#4	**#3 NOT (‘conference abstract’/it OR ‘conference review’/it)**	414
#3	**#1 AND #2**	542
#2	**‘patient attitude’/exp OR complian*:ab,ti,kw OR adheren*:ab,ti,kw OR noncomplian*:ab,ti,kw OR nonadheren*:ab,ti,kw OR dropout*:ab,ti,kw OR ‘drop-out*’:ab,ti,kw**	914,644
#1	**‘mandibular advancement’/exp OR ‘maxillomandibular advancement’/exp OR ‘sleep apnea device’/exp OR ((‘mandibular advancement’:ab,ti,kw OR ‘maxillomandibular advancement’:ab,ti,kw OR ‘mandibular reposition’:ab,ti,kw) AND (device*:ab,ti,kw OR appliance*:ab,ti,kw)) OR ‘protrusive device*’:ab,ti,kw OR 'protrusion device*':ab,ti,kw OR ‘protrusive appliance*’:ab,ti,kw OR ‘protrusion appliance*’:ab,ti,kw**	2579

**Wiley/Cochrane Library session results (21 Feb 2023)**

**Table:**

Search	Query	Items found
#3	**#1 AND #2**	139
#2	**(complian* OR adheren* OR noncomplian* OR nonadheren* OR dropout* OR (drop NEXT out*)):ab,ti,kw**	99,299
#1	**((((mandibular NEXT advancement) OR (maxillomandibular NEXT advancement) OR (mandibular NEXT reposition)) AND (device* OR appliance*)) OR (protrusive NEXT device*) OR (protrusion NEXT device*) OR (protrusive NEXT appliance*) OR (protrusion NEXT appliance*)):ab,ti,kw**	392

**Web of Science (core collection) session results (21 Feb 2023)**

**Table:**

Search	Query	Items found
#3	**#1 AND #2**	273
#2	**TS=(“complian*” OR “adheren*” OR “noncomplian*” OR “nonadheren*” OR “dropout*” OR “drop-out*”)**	509,091
#1	**TS=(((“mandibular advancement” OR “maxillomandibular advancement” OR “mandibular reposition”) AND (“device*” OR “appliance*”)) OR “protrusive device*” OR “protrusion device*” OR “protrusive appliance*” OR “protrusion appliance*”)**	1476
